# Detection of MAGE-A3 in breast cancer patients’ sentinel lymph nodes

**DOI:** 10.1054/bjoc.2001.2079

**Published:** 2001-09-01

**Authors:** R A Wascher, P J Bostick, K T Huynh, R Turner, K Qi, A E Giuliano, D S B Hoon

**Affiliations:** Department of Molecular Oncology, Joyce Eisenberg-Keefer Breast Center, Department of Pathology, Division of Biostatistics, John Wayne Cancer Institute, Saint John's Health Center, Santa Monica, CA, 90404

**Keywords:** breast cancer, MAGE-A3, micrometastases, RT-PCR, sentinel lymph node

## Abstract

The detection of occult metastatic breast cancer cells by RT-PCR is limited by the poor specificity of most tumour mRNA markers. MAGE-A3 is a highly specific tumour mRNA marker that is not expressed in non-cancer cells. This study assesses MAGE-A3 mRNA as a molecular marker for the detection of tumour cells in the sentinel lymph nodes (SLN) of breast cancer patients. Serial frozen sections of SLN (*n*= 121) were obtained from 77 AJCC (American Joint Committee on Cancer) Stage I–IIIA breast cancer patients. MAGE-A3 mRNA analysis of SLN was performed by RT-PCR and Southern blot analysis. Tumour cells were detected in 48 of 121 (40%) SLN from 77 patients by H&E or IHC staining, and 35 of 77 (45%) patients, overall, had histopathologically (H&E and/or IHC) positive SLN. Among histopathologically negative SLN, 28 of 73 (38%) SLN were MAGE-A3 mRNA positive by RT-PCR. Overall, 41 of 77 (53%) patients and 50 of 121 (41%) SLN were positive for MAGE-A3. MAGE-A3 mRNA expression in the SLN occurred more frequently with infiltrating lobular carcinoma (*P*< 0.001) than with infiltrating ductal carcinoma, adding further evidence of possible phenotypic differences between these 2 subtypes of breast cancer. Due to its high specificity, MAGE-A3 mRNA is a potentially useful marker for detecting breast cancer cells in the SLN. One half of breast tumours expressed MAGE-A3 mRNA, which has important potential implications for antigen-specific targeted immunotherapy. © 2001 Cancer Research Campaign


British Journal of Cancer (2001) **85**(9), 1340–1346
© 2001 Cancer Research Campaign
doi: 10.1054/ bjoc.2001.2079, available online at http://www.idealibrary.com on IDEAL

http://www.bjcancer.com

### Detection of MAGE-A3 in breast cancer patients' sentinel lymph nodes

**RA Wascher[1], PJ Bostick[1,2], KT Huynh[1], R Turner[3], K Qi[4], AE Giuliano[2] and DSB Hoon[1]**

[1]Department of Molecular Oncology, [2]Joyce Eisenberg-Keefer Breast Center, [3]Department of Pathology, and the [4]Division of Biostatistics, John Wayne Cancer Institute, Saint John's Health Center, Santa Monica, CA, 90404

**Summary** The detection of occult metastatic breast cancer cells by RT-PCR is limited by the poor specificity of most tumour mRNA markers. MAGE-A3 is a highly specific tumour mRNA marker that is not expressed in non-cancer cells. This study assesses MAGE-A3 mRNA as a molecular marker for the detection of tumour cells in the sentinel lymph nodes (SLN) of breast cancer patients. Serial frozen sections of SLN ($n$ = 121) were obtained from 77 AJCC (American Joint Committee on Cancer) Stage I–IIIA breast cancer patients. MAGE-A3 mRNA analysis of SLN was performed by RT-PCR and Southern blot analysis. Tumour cells were detected in 48 of 121 (40%) SLN from 77 patients by H&E or IHC staining, and 35 of 77 (45%) patients, overall, had histopathologically (H&E and/or IHC) positive SLN. Among histopathologically negative SLN, 28 of 73 (38%) SLN were MAGE-A3 mRNA positive by RT-PCR. Overall, 41 of 77 (53%) patients and 50 of 121 (41%) SLN were positive for MAGE-A3. MAGE-A3 mRNA expression in the SLN occurred more frequently with infiltrating lobular carcinoma ($P < 0.001$) than with infiltrating ductal carcinoma, adding further evidence of possible phenotypic differences between these 2 subtypes of breast cancer. Due to its high specificity, MAGE-A3 mRNA is a potentially useful marker for detecting breast cancer cells in the SLN. One half of breast tumours expressed MAGE-A3 mRNA, which has important potential implications for antigen-specific targeted immunotherapy. © 2001 Cancer Research Campaign

**Keywords**: breast cancer; MAGE-A3; micrometastases; RT-PCR; sentinel lymph node

Breast cancer patients who have undergone curative surgery and show no evidence of disease in the axillary lymph nodes or at distant sites have a recurrence rate as high as 30–40% over 5 to 20 years (Giuliano et al, 1994, 1997; Hellman, 1994). Currently, primary tumour pathologic factors are more useful in predicting which patients are at potential risk of regional lymph node metastases rather than those who will go on to develop systemic disease (Giuliano et al, 1994, 1995; Barth et al, 1997). The John Wayne Cancer Institute pioneered the SLN dissection procedure for breast cancer, and has demonstrated that the SLN accurately predicts the histopathologic status of the axillary basin in breast cancer patients, thereby allowing a more focused histopathological analysis (Giuliano et al, 1994, 1995, 1997).

If the SLN does not contain metastases by haematoxylin and eosin staining (H&E) or by immunohistochemistry staining (IHC), the chance of a non-SLN from the same axillary basin containing breast cancer cells, by H&E or IHC alone, is less than 1% (Giuliano et al, 1997). Serial sectioning of the SLN, along with examination by IHC and reverse transcriptase-polymerase chain reaction (RT-PCR), provides a powerful tool for improving staging of the axillary basin in breast cancer patients.

RT-PCR has previously been used as a molecular approach to the detection of occult metastatic tumour cells in the axillary lymph nodes, bone marrow, and blood of breast cancer patients (Datta et al, 1994; Schoenfeld et al, 1994; Burchill et al, 1995; Hoon et al, 1995a; Mori et al, 1995; Noguchi et al, 1996; Novaes et al, 1997; Yun et al, 1997; Zippelius et al, 1997; Bostick et al, 1998a). Previous reports have demonstrated the detection of occult metastases in lymph nodes by RT-PCR using carcinoembryonic antigen (CEA), cytokeratin-19 (CK-19), or mucin-1 (MUC-1) as messenger RNA (mRNA) markers (Schoenfeld et al, 1994; Mori et al, 1995; Noguchi et al, 1996; Bostick et al, 1998a). However, it has been shown, by our laboratory and others, that CEA, CK-19, and MUC-1 mRNA transcripts are present in normal epithelial cells, non-cancer cells of blood, normal lymph nodes, and normal bone marrow (Bostick et al, 1998a, 1998b, 1998c; Brugger et al, 1999; Lambrechts et al, 1999; Moinfar et al, 1999; Zeng et al, 1999; Xu et al, 2000). The rather frequent expression of these markers by non-cancer cells limits the potential clinicopathological utility and tumour specificity of these mRNA markers for detecting occult breast cancer micrometastasis (Schoenfeld et al, 1994; Hoon et al, 1995a; Bostick et al, 1998a).

The ideal mRNA tumour marker is one that is specifically expressed in cancer cells, but not in the non-cancer cells of the tissue or fluid being assessed. The *MAGE-A3* gene is a unique tumour mRNA marker in that it is not expressed in normal tissues except in the placenta and male germ cells (van der Bruggen et al, 1991; Gaugler et al, 1994; Chi et al, 1997; Bostick et al, 1999). The *MAGE-A3* gene belongs to a family of more than 12 closely related genes (*MAGE-A*) located on chromosome X (Oaks et al, 1994). Moreover, the *MAGE-A* gene proteins and peptides are immunogenic, and can induce cytotoxic T lymphocytes and antibodies in patients (van der Bruggen et al, 1991; Gaugler et al, 1994; Hoon et al, 1995c; Okamoto et al, 1997). Although originally identified as a melanoma antigen, the *MAGE-A3* gene is commonly expressed in various tumours of epithelial origin, including breast, lung, and colorectal carcinomas (van der Bruggen et al, 1991; Gaugler et al, 1994; Russo et al, 1995; Mori et al, 1996; Fujie et al, 1997; Bostick et al, 1999).

*Received 1 December 2000*
*Revised 16 July 2001*
*Accepted 23 July 2001*

*Correspondence to:* DSB Hoon

Earlier diagnosis of breast cancer, and prediction of recurrence, are 2 critical clinical interventions that may help prolong overall survival. Breast cancer is known to frequently metastasize to the regional tumour-draining lymph nodes (Giuliano et al, 1994; Hellman, 1994; Barth et al, 1997). Additionally, it is well documented that breast cancer cells spread haematogenously to bone marrow and other organs (Giuliano et al, 1994; Hellman, 1994). However, more than 30% of breast cancer patients with no evidence of disease metastatic to their lymph nodes, or to other distant sites at the time of initial diagnosis, will subsequently relapse and die of their disease. These patients are assumed to have subclinical disease that was undetected in the axillary nodes, and/or to have systemic disease that was otherwise undetected at the time of presentation. A molecular assay that can aid in the detection of occult metastatic cells in the tumour-draining lymph nodes may identify a sub-group of patients at high risk of systemic disease relapse, and may possibly be used for stratification of these patients into adjuvant therapy protocols. Moreover, the confirmation of a specific tumour's expression of immunogenic molecular markers, such as MAGE-A3, may identify patients who might benefit from targeted adjuvant immunotherapy. To this end, a reliable, sensitive and specific RT-PCR assay could help to improve staging of the axillary basin. The main objective of this study was to assess MAGE-A3 mRNA as a new molecular marker of micrometastatic breast cancer in SLN of breast cancer patients. Although MAGE-A3 has previously been detected in primary breast cancer, detection of breast cancer metastatic to the lymph nodes with MAGE-A3 has not been studied in large groups of patients (Russo et al, 1995).

### MATERIALS AND METHODS

### Sentinel node frozen section preparation

Surgical specimens were obtained after informed consent in consultation with the surgeon and pathologist. All tissues were collected and dissected under stringent sterile conditions to prevent RNA contamination. SLN were obtained from 77 consecutive AJCC Stage I–IIIA breast cancer patients undergoing SLN dissection, as previously described (Giuliano et al, 1994, 1995, 1997). Among these 77 patients, 60 were diagnosed with infiltrating ductal carcinoma (IDC), 10 with infiltrating lobular carcinoma (ILC), and 7 with other breast cancer variants. Primary tumour samples from these same 77 patients were not obtained, as most had very early stage disease, and many were referred following initial biopsy at other institutions. Institutional review board approval was obtained for human subject usage of lymph nodes obtained from all patients. SLN were prepared for histopathological examination, RT-PCR and Southern blot analysis as depicted in Figure 1. Each SLN was bisected, and an imprint slide was prepared from the tissue surface, and then stained with Diff-Quick I & II (Dade International, Miami, FL) (Reyes et al, 1998). An 8-µm section of tissue from the bisected frozen SLN was also stained with Diff-Quik, and used in the pathologist's intraoperative evaluation. 6 consecutive adjacent frozen sections were then cut on the cryostat, to a thickness of 12 µm each, and immediately stored at –80˚C until used in the RT-PCR assay. An additional 8-µm frozen section of tissue was then examined by Diff-Quik staining. If tumour cells were identified in

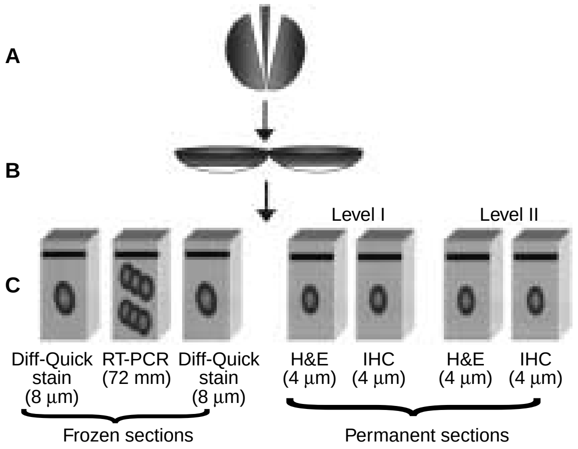

**Figure 1** Schematic diagram of SN preparation for histopathology and RT-PCR analysis: (**A**) Bisecting of SN with scalpel; (**B**) Sectioning frozen bisected SN; (**C**) Frozen sections serially cut for different assays the SLN by Diff-Quik staining, a complete axillary lymph node dissection was then performed. The bisected node was then placed in 10% formalin, embedded in a paraffin block, and a 4-µm-thick section was examined by light microscopy following H&E staining. If no metastases were identified by H&E staining alone, an adjacent 4-µm section was evaluated with anti-cytokeratin IHC. Anti-cytokeratin IHC was performed with a cocktail containing antibodies (MAK-6; Ciba-Corning, Alameda, CA) recognizing cytokeratins (CK)-8, 14, 15, 16, 18 and 19, using an automated immunoperoxidase staining processor (Ventana ES; Ventana Medical Systems, Inc, Tucson, AZ) (Turner et al, 1997, 1999). This permanent section evaluation was performed at 2 different levels, separated by approximately 40 µm. The portion of the SLN remaining after this extensive analysis was preserved in the event that further histopathological examination was necessary.

### RNA-positive and -negative controls

Six breast cancer cell lines were used as positive controls. The breast cancer cell lines MCF-7, BT-20 and MDA-MB-231 were obtained from the ATCC (Rockville, MD) and cultured according to the manufacturer's instructions. The 734B line is an established subclone of MCF-7. The cell lines JWCI BM-1 and JWCI JM-1 were established from primary invasive ductal carcinoma and characterized as breast cancer cell lines at JWCI (Hoon et al, 1996; Bostick et al, 1998c). All 6 cell lines were grown in RPMI 1640 (Gemini Bioproducts, Calabasas, CA) plus 10% fetal calf serum (heat inactivated), penicillin and streptomycin (GIBCO, Grant Island, NY), in culture flasks and passaged as previously described (Bostick et al, 1998a).

Lymph nodes obtained from 10 patients with benign surgical diseases were confirmed to be tumour-free by H&E staining and light microscopy, and were used for RT-PCR analysis as negative controls. 10 ml of blood collected from healthy donor volunteers in sodium citrate-containing tubes, as previously described, were also used as negative controls for the RT-PCR assay (Bostick et al, 1998a). The blood was centrifuged using a hypotonic density gradient solution, and nucleated cells (peripheral blood mononuclear cells; PBMC) in the blood were collected for RNA isolation as previously described (Bostick et al, 1998a).

*© 2001 Cancer Research Campaign* 

### RNA preparation

TRI-REAGENT (Molecular Research Center, Cincinnati, OH) was used to isolate total RNA from the cell lines, frozen section SLN, non-sentinel nodes, normal lymph nodes and blood following the manufacturer's instructions. RNA was then precipitated after adding 500 µl of isopropanol followed by storage at –30˚C for 12 h. The sample tube was then centrifuged at 14 000 g at 4˚C for 10 min. The sample was then washed with 75% ethanol, vacuum-dried, and resuspended in 10 mM Tris-HCl with 0.1 mM EDTA solution (pH 8). The concentration, purity and amount of total RNA were then determined by UV spectrophotometry. The integrity of all RNA samples was verified by performing RT-PCR with the housekeeping gene porphobilinogen deaminase (PBGD), and was confirmed by ethidium bromide gel electrophoresis (Bostick et al, 1999). If a sample did not express PBGD mRNA, the RNA was considered inadequate and no further analysis was performed. Tissue processing, RNA extraction, RT-PCR assay set-up, and post-PCR product analysis were carried out in separate designated rooms and buildings to prevent cross-contamination as previously reported (Bostick et al, 1998a, 1999).

### Oligonucleotide primers and probes

The primer sequence for MAGE-A3 was as follows: 5′ primer: 5′-GAA GCC GCC CCA GGC TCG-3′ and 3′ primer: 5′-GGA GTC CTC ATA GGA TTG GCT CC-3′. The MAGE-A3 RT-PCR cDNA product produced with this primer set was 423 base pairs in size (Hoon et al, 1995b). Purified fragments of the cDNA products amplified with MAGE-A3 primer sets were labelled with digoxigenin (Boehringer Mannheim, La Jolla, CA) and used as DNA probes in the Southern blot assay (Bostick et al, 1998a). The PBGD primers have been previously described (Finke et al, 1993).

### RT-PCR assay

The RT-PCR assay was carried out as previously described (Bostick et al, 1999). RT was performed on 1 µg of total RNA for each RT reaction, as specified for Moloney murine leukaemia virus-reverse transcriptase (Promega, Madison, WI). The RNA was incubated at 70˚C for 5 min and then put on ice before addition of RT reaction reagents. Molecular biology grade water was used in place of RNA as a negative control for all RT reactions. The RNA with RT reagents added was incubated at 37˚C for 2 h, followed by heating at 95˚C for 5 min. All RT reactions were carried out with oligo-dT priming. The PCR conditions were as follows: 1 cycle of denaturing at 95˚C for 5 min, followed by 35 cycles of 95˚C for 1 min, 55˚C for 1 min, and 72˚C for 1 min before a final extension at 72˚C for 10 min. RT-PCR optimal conditions were accomplished using an Omni thermocycler (Hybaid, Middlesex, UK). The RT-PCR cDNA products run on 2% agarose gel were alkaline-denatured, and Southern blotting was performed as previously described (Bostick et al, 1998a). The MAGE-A3 cDNA probe was developed from the RT-PCR cDNA products and labelled with digoxigenin. Specific binding was detected using anti-digoxigenin, alkaline phosphatase-conjugated antibody (Boehringer Mannheim, Indianapolis, IN) as described by the manufacturer. Any discrepancies or faint signals in Southern blotting were repeated for verification. In each experiment set-up, samples of RT-PCR reagents without mRNA, and previously tested mRNA marker-negative healthy donor blood cells and lymph nodes were used as negative controls. Previously tested positive tumour cell lines or tumour biopsy specimens were included in the assay as positive RT-PCR and Southern blot controls. If known reagent control samples were different than expected, the results were discarded and the assay was repeated with new reagents. Results in the analysis of all specimens were determined to be positive only after verification by Southern blotting.

### RESULTS

### MAGE-A3 mRNA expression in specimens

Breast cancer cell lines, primary breast cancer tumours and blood and lymph nodes from healthy volunteers were used as RT-PCR controls. 4 of 6 (67%) breast cancer cell lines and 5 of 10 (50%) primary breast cancers assessed expressed MAGE-A3 mRNA (Table 1). A positive result was indicated by a 423-bp size cDNA band detected by RT-PCR and Southern blotting (referred from this point as RT-PCR assay) (Figure 2). Donor blood cells from 25 healthy volunteers (15 females) were evaluated for MAGE-A3 mRNA marker expression. MAGE-A3 mRNA was not detected in any of the donor blood cells from these healthy volunteers. After informed consent, normal lymph nodes (confirmed histopathologically) were obtained from 10 patients undergoing non-cancer surgery (e.g., cholecystectomy, hernia repair). MAGE-A3 mRNA was not expressed in any of these normal lymph nodes. This high frequency of MAGE-A3 mRNA expression in breast cancer tumour cells, and the lack of expression in normal controls, led us to further investigate its clinicopathologic utility for the detection of metastatic cells in the SLN of breast cancer patients.

### Sensitivity of RT-PCR assay

Total RNA was isolated from breast carcinoma cell lines and serially diluted for the RT-PCR assay to determine its sensitivity. An in

**Table 1** MAGE-A3 mRNA marker expression[a]

| RNA source | *n* | MAGE-A3 mRNA marker expression (%) |
|---|---|---|
| Breast cancer cell lines | 6 | 4 (67) |
| Primary breast tumours | 10 | 5 (50) |
| Normal non-cancer donor bloods | 25 | 0 |
| Normal non-cancer lymph nodes | 10 | 0 |

[a]mRNA marker expression refers to RT-PCR and Southern blot analysis (all specimens assessed were mRNA-positive for PBGD by ethidium bromide gel analysis).

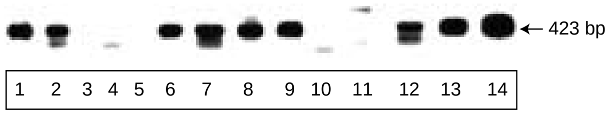

**Figure 2** Representative examples of MAGE-A3 mRNA expression by the RT-PCR assay. Lanes 1 and 2 correspond to primary breast tumours; lane 3, $H_2O$ (negative control); lanes 4 and 5, normal PBL (negative controls); lanes 6–10, representative frozen sections of SN; lanes 11 and 12, non-SLN; and lanes 13 and 14, breast cancer cell lines MCF-7 and JWCI JM-1, respectively. All RT-PCR assays were performed with 1 µg RNA, and cDNA products were equally loaded on the gel for Southern blotting. All specimens assessed were mRNA positive for PBGD by ethidium bromide gel electrophoresis analysis.

 *© 2001 Cancer Research Campaign*

**Table 2** MAGE-A3 mRNA expression and histopathology of SN

| SN histopathology by H&E/IHC[a] | Number of SN | RT-PCR positive for MAGE-A3 (%) |
|---|---|---|
| H&E (+) | 33 | 15 (45) |
| H&E (–) | 88 | 35 (40) |
| H&E (–)/IHC (–) | 73 | 28 (38) |
| H&E (–)/IHC (+) | 15 | 7 (47) |

[a]H&E/IHC (+) refers to positive for either H&E staining and/or IHC analysis; H&E/IHC (–) refers to negative for both methods of histopathological analysis.

vitro model was assessed whereby serially diluted (1 to $10^5$) breast cancer cells (MCF-7) were mixed with $10^7$ normal donor PBMC. The detection sensitivity of the assay was approximately 1–5 breast cancer cells in $10^7$ PBMC (data not shown).

### Histopathology of sentinel lymph nodes

The SLN ($n$ = 121) from 77 AJCC stage I–IIIA breast cancer patients undergoing SLND were analysed by H&E. Nodes that appeared negative by H&E evaluation for tumour cells were then assessed by IHC (Table 2). In 48 of 121 (40%) SLN, tumour cells were detected by histopathological means (H&E or IHC) and 35 of 77 (45%) patients had histopathologically positive SLN. The remaining 73 of 121 (60%) SLN did not contain detectable tumour cells by histopathological analysis (H&E or IHC). MAGE-A3 mRNA was expressed in 22 of 48 (46%) SLN that were positive for tumour by H&E or IHC (Table 2). MAGE-A3 mRNA was also detected in 28 of 73 (38%) histopathologically negative (by H&E or IHC) SLN. Overall, 41 of 77 (53%) of patients and 50 of 121 (41%) SLN were positive for tumour cells by MAGE-A3 mRNA expression.

### Correlation of MAGE-A3 mRNA expression with prognostic factors

MAGE-A3 mRNA expression in the SLN was evaluated for correlation with currently accepted prognostic factors for breast carcinoma. MAGE-A3 mRNA was expressed in the SLN of 10 of 10 (100%) patients with ILC compared to only 30 of 60 (50%) SLN in patients with IDC, which was significant ($P < 0.001$, Fisher's exact test). The mean Bloom–Richardson score was 6.5 ± 1.6 for SLN that expressed MAGE-A3 mRNA compared to 5.6 ± 1.6 for SLN that did not express MAGE-A3 mRNA, and approached significance ($P = 0.07$). The SLN of 12 of 17 (71%) patients with poorly differentiated tumours expressed MAGE-A3 mRNA compared with the SLN of 4 of 12 (33%) patients with well-differentiated tumours ($P = 0.13$). This tumour grade correlation with MAGE-A3 expression did not reach statistical significance, most likely due to the small sample size of patients with either poorly or well differentiated tumours. 9 of 12 (75%) SLN from oestrogen receptor (ER)-negative primary tumours expressed MAGE-A3, while 30 of 62 (48%) SLN from ER-positive tumours expressed MAGE-A3, though this association also did not quite reach statistical significance ($P = 0.9$). Age, menopausal status, family history, tumour size, oestrogen receptor status, progesterone receptor status, S-phase content, tumour ploidy, HER-2/neu status, intraductal extent of tumour, and angiolymphatic invasion did not appear to correlate with the MAGE-A3 RT-PCR status of the SLN.

### DISCUSSION

The ideal mRNA tumour marker for RT-PCR evaluation is one that is expressed frequently in cancer cells, but not in non-cancer cells of the tissue/fluid compartment being assessed. The detection of micrometastatic breast cancer cells by RT-PCR analysis has been limited by the lack, thus far, of mRNA markers specific to breast carcinoma. In this study, the MAGE-A3 mRNA marker was assessed as a candidate molecular marker for detecting breast cancer in SLN because of its high degree of specificity for breast cancer cells, and its absence of expression in normal cells in the breast, lymph nodes and blood (Fujie et al, 1997).

During conventional H&E histopathological evaluation of lymph nodes, the nodes are cut into a limited number of sections prior to microscopic evaluation. There are, however, several methods of increasing the histopathological yield of lymph nodes. The yield of tumour cell detection in the axillary lymph nodes can be increased over standard H&E histopathological evaluation by as much as 33% merely by cutting multiple thin serial sections (International (Ludwig) Breast Cancer Study Group, 1990; Neville et al, 1992).

At present, there is considerable controversy regarding the reproducibility and accuracy of IHC, as well as the clinical significance of IHC-positive lymph nodes draining the primary tumour site (Carter et al, 2000; Fitzgibbons et al, 2000; Hammond et al, 2000). These concerns have been amplified by the recent finding that benign epithelial cells can be identified in the axillary lymph nodes of patients following breast biopsy, and which appear to be translocated to the node following surgical manipulation of the breast during initial biopsy (Carter et al, 2000). Irrespective of the debate about the accuracy and reproducibility of IHC, the presence of such benign mammary epithelial cells in the axillary nodes of a patient undergoing breast cancer surgery would likely be interpreted as occult tumor cell(s) by IHC assays against cytokeratins. Thus the use of MAGE-A3 RT-PCR, given that MAGE-A3 is not expressed in normal mammary epithelial cells, may increase the specificity and accuracy of evaluating SLN for micrometastases over IHC, as IHC stains for cytokeratins will demonstrate a positive result for both benign and malignant epithelial cells. Additionally, RT-PCR of processed SLN tissue may obviate the potential for false negatives that occur due to sampling errors during conventional histopathological processing and analysis.

In our study, 33 of 121 (27%) SLN were identified as positive for tumour cells by H&E, and 15 (45%) of these 33 SLN were positive for MAGE-A3 expression. SLN that were H&E negative accounted for 88 of 121 (73%), and 35 of these 88 (40%) H&E-negative SLN were RT-PCR positive for MAGE-A3. This is in contrast to the 15 (17%) of 88 H&E-negative SLN upstaged by IHC alone in this study. A total of 15 of 121 (12%) SLN were H&E-negative and IHC-positive, and 7 of these 15 (47%) SLN were positive for MAGE-A3. Among SLN histopathologically negative by both H&E and IHC, 28 of 73 (38%) were positive based upon MAGE-A3 mRNA expression.

The direct comparison of RT-PCR results and standard histopathological results (e.g., H&E and IHC) for the same tumour specimens can be problematic. The expression of specific tumour-associated mRNA markers is heterogeneous within a population of primary or metastatic cancer cells. This factor accounts for the commonly observed pattern of expression of individual specific tumour markers in a population of tumour cells, whereby only a subset of cells derived from primary and metastatic tumours will

© 2001 Cancer Research Campaign express such markers. Thus the presence or absence of tumour-associated molecular markers does not always directly correspond to the standard histopathological features used to confirm tumour cell presence.

Patients with micrometastases detected by histopathological and/or RT-PCR analysis may represent a subgroup at increased risk of disease relapse. Approximately 30–40% of patients with no histopathological evidence of metastases to the axillary basin will subsequently relapse with distant disease (Giuliano et al, 1994, 1995; Hellman, 1994). This may in part be due to undetected micrometastatic disease in lymph nodes, haematogenous spread, and/or clinically undetectable disseminated disease at the time of presentation. At the same time, patients who have histopathologically positive SLN do not all go on to develop disseminated disease, as individual tumour cell characteristics and host factors likely play a crucial role in deterring circulating tumour cells from forming successfully established metastatic tumours. Long-term prospective studies should determine if breast cancer patients with lymph node micrometastases, detected by H&E, IHC or by RT-PCR molecular assays, are at increased risk of subsequent disease relapse. The American College of Surgeons Oncology Group Z0010 trial (PI: Armando Giuliano, JWCI) is currently underway, and should provide meaningful answers to such questions within the next 5–10 years.

In terms of the prognostic significance of MAGE-A3 expression in the SLN of breast cancer patients, we have recently demonstrated that the presence of MAGE-A3 in the SLN of patients with melanoma, combined with other melanoma RT-PCR mRNA markers, significantly correlated with a greater risk of disease recurrence (Bostick et al, 1999). If breast cancer SLN micrometastases possess the same prognostic correlation with disseminated disease as appears to be the case with melanoma, then the detection of MAGE-A3 in the histopathologically negative SLN of breast cancer patients may prove to be of significant prognostic importance as well. As a tertiary referral centre for breast cancer, 73 of the 77 (95%) of our study patients had AJCC stage I or II breast cancer at the time of inclusion in the study, with a mean follow-up of just over 5 years to date. In view of the frequently prolonged natural history of breast cancer metastasis, meaningful prognostic correlation of clinical outcome with MAGE-A3 SLN status cannot be addressed by this study at this time. However, as of June 2001, follow-up information was available for 65 of the 76 (86%) patients who underwent resection of their primary tumours and SLN ≥ 5 years ago. Of these 65 patients, one has developed spinal metastasis (AJCC stage I at initial diagnosis) and her SLN was positive for MAGE-A3. A second patient is currently pending biopsy for a highly suspicious breast ultrasound obtained at follow up, and her SLN was MAGE-A3 positive. A third patient is presently being evaluated for a newly elevated CA 27.29 level (stage IIA at initial diagnosis), but she is without clinically or radiographically evident disease, and her SLN was negative for MAGE-A3. The remaining 62 patients are presently without clinical, radiographic or laboratory evidence of disease at this time.

An interesting finding in this study was that expression of MAGE-A3 in the SLN was significantly more common in patients with ILC than in IDC ($P < 0.001$, Fisher's exact test). SLN expression of MAGE-A3 was also correlated with a higher Bloom–Richardson score, approaching statistical significance ($P = 0.07$, Wilcoxon rank sums test). This finding is consistent with our previous studies which have demonstrated that tumours with higher Bloom–Richardson (BR) scores are at greater risk of relapse than tumours receiving low BR scores (Bostick et al, 1998c). Although the overall clinical prognosis is similar for both ILC and IDC, there is a known increased risk of bilateral and multifocal disease with ILC. Moreover, there are recent data that suggest that true histopathologic, genetic and clinical prognostic differences exist between ILC and IDC. For example, there is evidence that ILC variants may have a different prognostic outcome as compared to IDC (Toikkanen et al, 1997; Bentz et al, 1998; Sinha et al, 2000). Distinctive patterns of chromosomal imbalance may also be found when comparing ILC to IDC (Richard et al, 2000), as well as histopathological differences (Lee et al, 1998; Prasad et al, 1998). The findings of our study may be further evidence that significant phenotypic differences exist between these 2 invasive breast cancer variants, despite their otherwise similar clinical outcomes.

In view of the known immunogenicity of MAGE antigens, patients with breast tumours that express MAGE-A3 may potentially benefit from active-specific immunotherapy targeted to MAGE-A3 protein epitopes. Active-specific immunotherapies used to date are in the form of vaccines with MAGE-A3 peptides, *MAGE-A3* gene vaccines, or dendritic cells pulsed with MAGE-A3 peptide (Okamoto et al, 1997; Thurner et al, 1999; Weber et al, 1999). Post-surgery adjuvant vaccination may be useful in patients who have MAGE-A3-expressing metastatic breast tumour cells to reduce the incidence of disease recurrence, or to control existing subclinical disease progression. Adjuvant vaccine therapy in high-risk clinically disease-free patients provides a non-toxic maintenance therapy that may prolong or prevent disease recurrence (Pantel et al, 1999). Thus, MAGE-A3 RT-PCR assay for SLN may find relevance in the clinic in several ways, to include the monitoring of patients during treatment; the identification of patients likely to benefit from active-specific immunotherapy with MAGE-A3 related products; and/or the assessment of patients at potentially high risk for disease recurrence.

In terms of future investigation, a logical next step would be to compare the expression of MAGE-A3 in both the primary tumours and their associated SLN. The continued follow up of our study patients may also show a correlation between SLN positive for MAGE-A3 and clinical prognosis, which is not evident now at just 5 years of follow up in these stage I and II breast cancer patients. Additionally, future follow-up data from patients included in this study may also add important prognostic data that are not currently available.

In summary, we have shown that the MAGE-A3 mRNA RT-PCR assay may have a role in the detection of occult tumour cells in the SLN of breast cancer patients. The detection of this mRNA marker indicates a greater likelihood of micrometastatic disease presence, as it is expressed in at least 50% of breast cancer tumours, but not in benign mammary cells, lymph nodes or blood. Long-term follow up of patients with SLN positive for MAGE-A3 mRNA expression will yield additional important data regarding the correlation between the presence of this marker in the SLN and clinical prognosis. There is also intriguing evidence of differences in the expression frequency of MAGE-A3 between ILC and IDC tumours, which adds to the growing evidence that these 2 breast cancer subtypes may indeed be distinctive from each other in clinically significant respects. Finally, the finding that approximately one-half of breast tumours express MAGE-A3 may have value in stratifying patients to receive active-specific immunotherapy against MAGE-A3 epitopes in prospective trials, and to follow their responses to these and other therapies.

 *© 2001 Cancer Research Campaign*

### ACKNOWLEDGEMENTS

This study was supported in part by the Ben B and Joyce E Eisenberg Foundation, Los Angeles, CA; the Fashion Footwear Association of New York (FANY); grant DMD17-96-1-6193 from the United States Army; and the Gonda Breast Cancer Research Laboratories. We would like to thank the clinical staff of JWCI and the Joyce-Eisenberg Keefer Center.

### REFERENCES

Barth A, Craig PH and Silverstein MJ (1997) Predictors of axillary lymph node metastases in patients with T1 breast carcinoma. *Cancer* **79**: 1918–1922

Bentz JS, Yassa N and Clayton F (1998) Pleomorphic lobular carcinoma of the breast: clinicopathologic features of 12 cases. *Mod Pathol* **11**: 814–822

Bostick PJ, Chatterjee S, Chi DD, Huynh KT, Giuliano AE, Cote R and Hoon DS (1998a) Limitations of specific reverse-transcriptase polymerase chain reaction markers in the detection of metastases in the lymph nodes and blood of breast cancer patients. *J Clin Oncol* **16**: 2632–2640

Bostick PJ, Hoon DS and Cote RJ (1998b) Detection of carcinoembryonic antigen messenger RNA in lymph nodes from patients with colorectal cancer [letter; comment]. *N Engl J Med* **339**: 1643–1644

Bostick PJ, Huynh KT, Sarantou T, Turner RR, Qi K, Giuliano AE and Hoon DS (1998c) Detection of metastases in sentinel lymph nodes of breast cancer patients by multiple-marker RT-PCR. *Int J Cancer* **79**: 645–651

Bostick PJ, Morton DL, Turner RR, Huynh KT, Wang HJ, Elashoff R, Essner R and Hoon DS (1999) Prognostic Significance of Occult Metastases Detected by Sentinel Lymphadenectomy and Reverse Transcriptase-Polymerase Chain Reaction in Early-Stage Melanoma Patients. *J Clin Oncol* **17**: 3238–3244

Brugger W, Buhring HJ, Grunebach F, Vogel W, Kaul S, Muller R, Brummendorf TH, Ziegler BL, Rappold I, Brossart P, Scheding S and Kanz L (1999) Expression of MUC-1 epitopes on normal bone marrow: implications for the detection of micrometastatic tumor cells. *J Clin Oncol* **17**: 1535–1544

Burchill SA, Bradbury MF, Pittman K, Southgate J, Smith B and Selby P (1995) Detection of epithelial cancer cells in peripheral blood by reverse transcriptase-polymerase chain reaction. *Br J Cancer* **71**: 278–281

Carter BA, Jensen RA, Simpson JF and Page DL (2000) Benign transport of breast epithelium into axillary lymph nodes after biopsy. *Am J Clin Pathol* **113**: 259–265

Chi DD, Merchant RE, Rand R, Conrad AJ, Garrison D, Turner R, Morton DL and Hoon DS (1997) Molecular detection of tumor-associated antigens shared by human cutaneous melanomas and gliomas. *Am J Pathol* **150**: 2143–2152

Datta YH, Adams PT, Drobyski WR, Ethier SP, Terry VH and Roth MS (1994) Sensitive detection of occult breast cancer by the reverse-transcriptase polymerase chain reaction. *J Clin Oncol* **12**: 475–482

Finke J, Fritzen R, Ternes P, Lange W and Dolken G (1993) An improved strategy and a useful housekeeping gene for RNA analysis from formalin-fixed, paraffin-embedded tissues by PCR. *Biotechniques* **14**: 448–453

Fitzgibbons PL, Page DL, Weaver D, Thor AD, Allred DC, Clark GM, Ruby SG, O'Malley F, Simpson JF, Connolly JL, Hayes DF, Edge SB, Lichter A and Schnitt SJ (2000) Prognostic factors in breast cancer. College of American Pathologists Consensus Statement 1999. *Arch Pathol Lab Med* **124**: 966–978

Fujie T, Mori M, Ueo H, Sugimachi K and Akiyoshi T (1997) Expression of MAGE and BAGE genes in Japanese breast cancers. *Ann Oncol* **8**: 369–372

Gaugler B, Van den Eynde B, van der Bruggen P, Romero P, Gaforio JJ, De Plaen E, Lethe B, Brasseur F and Boon T (1994) Human gene MAGE-3 codes for an antigen recognized on a melanoma by autologous cytolytic T lymphocytes. *J Exp Med* **179**: 921–930

Giuliano AE, Kirgan DM, Guenther JM and Morton DL (1994) Lymphatic mapping and sentinel lymphadenectomy for breast cancer. *Ann Surg* **220**: 391–398

Giuliano AE, Dale PS, Turner RR, Morton DL, Evans SW and Krasne DL (1995) Improved axillary staging of breast cancer with sentinel lymphadenectomy. *Ann Surg* **222**: 394–399

Giuliano AE, Jones RC, Brennan M and Statman R (1997) Sentinel lymphadenectomy in breast cancer. *J Clin Oncol* **15**: 2345–2350

Hammond ME, Fitzgibbons PL, Compton CC, Grignon DJ, Page DL, Fielding LP, Bostwick D and Pajak TF (2000) College of American Pathologists Conference XXXV: solid tumor prognostic factors-which, how and so what? Summary document and recommendations for implementation. Cancer Committee and Conference Participants. *Arch Pathol Lab Med* **124**: 958–965

Hellman S (1994) Karnofsky Memorial Lecture: Natural history of small breast cancers. *J Clin Oncol* **12**: 2229–2234

Hoon DS, Doi F, Giuliano AE, Schmid P and Conrad AJ (1995a) The detection of breast carcinoma micrometastases in axillary lymph nodes by means of reverse transcriptase-polymerase chain reaction [letter]. *Cancer* **76**: 533–535

Hoon DS, Wang Y, Dale PS, Conrad AJ, Schmid P, Garrison D, Kuo C, Foshag LJ, Nizze AJ and Morton DL (1995b) Detection of occult melanoma cells in blood with a multiple-marker polymerase chain reaction assay. *J Clin Oncol* **13**: 2109–2116

Hoon DS, Yuzuki D, Hayashida M and Morton DL (1995c) Melanoma patients immunized with melanoma cell vaccine induce antibody responses to recombinant MAGE-1 antigen. *J Immunol* **154**: 730–737

Hoon DS, Sarantou T, Doi F, Chi DD, Kuo C, Conrad AJ, Schmid P, Turner R and Guiliano A (1996) Detection of metastatic breast cancer by beta-hCG polymerase chain reaction. *Int J Cancer* **69**: 369–374

International (Ludwig) Breast Cancer Study Group (1990) Prognostic importance of occult axillary lymph node micrometastases from breast cancers. *Lancet* **335**: 1565–1568

Lambrechts AC, Bosma AJ, Klaver SG, Top B, Perebolte L, van't Veer LJ and Rodenhuis S (1999) Comparison of immunocytochemistry, reverse transcriptase polymerase chain reaction, and nucleic acid sequence-based amplification for the detection of circulating breast cancer cells. *Breast Cancer Res Treat* **56**: 219–231

Lee AH, Dublin EA, Bobrow LG and Poulsom R (1998) Invasive lobular and invasive ductal carcinoma of the breast show distinct patterns of vascular endothelial growth factor expression and angiogenesis. *J Pathol* **185**: 394–401

Moinfar F, Man YG, Lininger RA, Bodian C and Tavassoli FA (1999) Use of keratin 35betaE12 as an adjunct in the diagnosis of mammary intraepithelial neoplasia-ductal type—benign and malignant intraductal proliferations. *Am J Surg Pathol* **23**: 1048–1058

Mori M, Mimori K, Inoue H, Barnard GF, Tsuji K, Nanbara S, Ueo H and Akiyoshi T (1995) Detection of cancer micrometastases in lymph nodes by reverse transcriptase-polymerase chain reaction. *Cancer Res* **55**: 3417–3420

Mori M, Inoue H, Mimori K, Shibuta K, Baba K, Nakashima H, Haraguchi M, Tsuji K, Ueo H, Barnard GF and Akiyoshi T (1996) Expression of MAGE genes in human colorectal carcinoma. *Ann Surg* **224**: 183–188

Neville AM, Bettelheim R, Gelber RD, Save-Soderbergh J, Davis BW, Reed R, Torhorst J, Golouh R, Peterson HF, Price KN and et al (1992) Factors predicting treatment responsiveness and prognosis in node-negative breast cancer. The International (Ludwig) Breast Cancer Study Group. *J Clin Oncol* **10**: 696–705

Noguchi S, Aihara T, Motomura K, Inaji H, Imaoka S and Koyama H (1996) Detection of breast cancer micrometastases in axillary lymph nodes by means of reverse transcriptase-polymerase chain reaction. Comparison between MUC1 mRNA and keratin 19 mRNA amplification. *Am J Pathol* **148**: 649–656

Novaes M, Bendit I, Garicochea B and del Giglio A (1997) Reverse transcriptase-polymerase chain reaction analysis of cytokeratin 19 expression in the peripheral blood mononuclear cells of normal female blood donors. *Mol Pathol* **50**: 209–211

Oaks MK, Hanson JP, Jr. and O'Malley DP (1994) Molecular cytogenetic mapping of the human melanoma antigen (MAGE) gene family to chromosome region Xq27-qter: implications for MAGE immunotherapy. *Cancer Res* **54**: 1627–1629

Okamoto T, Kaneda Y, Yuzuki D, Huang SK, Chi DD and Hoon DS (1997) Induction of antibody response to human tumor antigens by gene therapy using a fusigenic viral liposome vaccine. *Gene Ther* **4**: 969–976

Pantel K, Cote RJ and Fodstad O (1999) Detection and clinical importance of micrometastatic disease. *J Natl Cancer Inst* **91**: 1113–1124

Prasad ML, Osborne MP and Hoda SA (1998) Observations on the histopathologic diagnosis of microinvasive carcinoma of the breast. *Anat Pathol* **3**: 209–232

Reyes CV, Thompson KS, Jensen JD and Choudhury AM (1998) Metastasis of unknown origin: the role of fine-needle aspiration cytology. *Diagn Cytopathol* **18**: 319–322

Richard F, Pacyna-Gengelbach M, Schlüns K, Fleige B, Winzer KJ, Szymas J, Dietel M, Petersen I and Schwendel A (2000) Patterns of chromosomal imbalances in invasive breast cancer. *Int J Cancer* **89**: 305–310

Russo V, Traversari C, Verrecchia A, Mottolese M, Natali PG and Bordignon C (1995) Expression of the MAGE gene family in primary and metastatic human breast cancer: implications for tumor antigen-specific immunotherapy. *Int J Cancer* **64**: 216–221

Schoenfeld A, Luqmani Y, Smith D, O'Reilly S, Shousha S, Sinnett HD and Coombes RC (1994) Detection of breast cancer micrometastases in axillary lymph nodes by using polymerase chain reaction. *Cancer Res* **54**: 2986–2990

Sinha PS, Bendall S and Bates T (2000) Does routine grading of invasive lobular cancer of the breast have the same prognostic significance as for ductal cancers? *Eur J Surg Oncol* **26**: 733–737

© 2001 Cancer Research Campaign

Thurner B, Haendle I, Roder C, Dieckmann D, Keikavoussi P, Jonuleit H, Bender A, Maczek C, Schreiner D, von den Driesch P, Brocker EB, Steinman RM, Enk A, Kampgen E and Schuler G (1999) Vaccination with mage-3A1 peptide-pulsed mature, monocyte-derived dendritic cells expands specific cytotoxic T cells and induces regression of some metastases in advanced stage IV melanoma. *J Exp Med* **190**: 1669–1678

Toikkanen S, Pylkkanen L and Joensuu H (1997) Invasive lobular carcinoma of the breast has better short-and long-term survival than invasive ductal carcinoma. *Br J Cancer* **76**: 1234–1240

Turner RR, Ollila DW, Krasne DL and Giuliano AE (1997) Histopathologic validation of the sentinel lymph node hypothesis for breast carcinoma. *Ann Surg* **226**: 271–276

Turner RR, Ollila DW, Stern S and Giuliano AE (1999) Optimal histopathologic examination of the sentinel lymph node for breast carcinoma staging [see comments]. *Am J Surg Pathol* **23**: 263–267

Van der Bruggen P, Traversari C, Chomez P, Lurquin C, De Plaen E, Van den Eynde B, Knuth A and Boon T (1991) A gene encoding an antigen recognized by cytolytic T lymphocytes on a human melanoma. *Science* **254**: 1643–1647

Weber JS, Hua FL, Spears L, Marty V, Kuniyoshi C and Celis E (1999) A phase I trial of an HLA-A1 restricted MAGE-3 epitope peptide with incomplete Freund's adjuvant in patients with resected high-risk melanoma. *J Immunother* **22**: 431–440

Xu X, Roberts SA, Pasha TL and Zhang PJ (2000) Undesirable cytokeratin immunoreactivity of native nonepithelial cells in sentinel lymph nodes from patients with breast carcinoma. *Arch Pathol Lab Med* **124**: 1310–1313

Yun K, Gunn J, Merrie AE, Phillips LV and McCall JL (1997) Keratin 19 mRNA is detectable by RT-PCR in lymph nodes of patients without breast cancer [letter; comment]. *Br J Cancer* **76**: 1112–1113

Zeng Z, Melamed J, Symmans PJ, Cangiarella JF, Shapiro RL, Peralta H and Symmans WF (1999) Benign proliferative nipple duct lesions frequently contain CAM 5.2 and anti-cytokeratin 7 immunoreactive cells in the overlying epidermis. *Am J Surg Pathol* **23**: 1349–1355

Zippelius A, Kufer P, Honold G, Kollermann MW, Oberneder R, Schlimok G, Riethmuller G and Pantel K (1997) Limitations of reverse-transcriptase polymerase chain reaction analyses for detection of micrometastatic epithelial cancer cells in bone marrow. *J Clin Oncol* **15**: 2701–2708

*© 2001 Cancer Research Campaign*
